# Low-Fluence Photodynamic Therapy versus Subthreshold Micropulse Yellow Wavelength Laser in the Treatment of Chronic Central Serous Chorioretinopathy

**DOI:** 10.1155/2016/3513794

**Published:** 2016-08-15

**Authors:** Emin Özmert, Sibel Demirel, Özge Yanık, Figen Batıoğlu

**Affiliations:** Department of Ophthalmology, Faculty of Medicine, Ankara University, 06620 Ankara, Turkey

## Abstract

*Purpose*. To compare the efficacy and safety of subthreshold micropulse yellow wavelength laser (SMYL) and low-fluence photodynamic therapy (PDT) in the treatment of chronic central serous chorioretinopathy (CSC).* Methods*. Thirty-three eyes of 30 patients with chronic CSC received either PDT (18 eyes) or SMYL (15 eyes) therapy. Best corrected visual acuity (BCVA), subretinal fluid (SRF) height, and central macular thickness (CMT) were evaluated at the baseline visit and one, three, six, nine, and 12 months after the therapy.* Results*. After 12 months, mean BCVA improved from 67.3 ± 14.2 to 71.5 ± 21.4 ETDRS letters in SMYL group and from 60.7 ± 16.3 to 64.4 ± 24.9 ETDRS letters in PDT group (*p* = 0.285 and *p* = 0.440, resp.). Mean CMT decreased from 242.8 ± 80 *μ*m to 156.9 ± 60 *μ*m in the PDT group and from 287.3 ± 126 *μ*m to 138.0 ± 40 *μ*m in the SMYL group (*p* = 0.098 and *p* = 0.003, resp.). SRF resolved completely in 72.2% and 80.0% of the eyes in the PDT and SMYL groups, respectively. Mean SRF height decreased from 117.2 ± 58 *μ*m to 31.3 ± 56 *μ*m in the PDT group and from 130.0 ± 104 *μ*m to 12.5 ± 21 *μ*m in the SMYL group (*p* = 0.031 and *p* = 0.014, resp.).* Conclusions*. Subthreshold micropulse yellow wavelength laser seems to be effective in the treatment of chronic CSC without any side effect and results in the resorption of SRF without causing visible retinal scarring.

## 1. Introduction

Central serous chorioretinopathy (CSC) is a disorder of unknown etiology characterized by detachment of the neurosensory retina due to accumulation of serous fluid between the retinal pigment epithelium (RPE) and photoreceptor layers. The disease can present in acute or chronic form. Acute CSC is generally self-limited with spontaneous regression, and it causes minimal sequelae. In chronic CSC, the long-term persistence (longer than six months) of subretinal fluid (SRF) can result in atrophy of the RPE, cystoid retinal degeneration, choroidal neovascularization, and permanent vision loss [1, 2].

Management of chronic CSC includes various options, such as risk factor modification (discontinuation of steroids) [3], medical treatment (carbonic anhydrase inhibitors) [4], conventional focal laser [5], photodynamic therapy (PDT) [6, 7], and intravitreal injection of vascular endothelial growth factor inhibitors [6, 8]. It has been reported that PDT with verteporfin induces the resorption of SRF by reducing choroidal vascular hyperpermeability [9, 10]. However, it has the potential for serious side effects, such as choroidal ischemia, RPE atrophy, and iatrogenic choroidal neovascularization [11, 12]. To avoid these complications, newer PDT protocols, including half-dose [13] and low-fluence [14] applications, have been developed.

Nowadays, micropulse laser (MPL) photocoagulation is another possible treatment option for chronic CSC [15, 16]. The mechanism of action depends on targeting a train of ultrashort laser pulses at the particular tissue of interest. These repetitive bursts prevent damage to adjacent tissues from thermal effects, minimize total energy use, and provide time for tissue cooling between pulses [17]. This technology can be paired with either 810 nm or 577 nm wavelength lasers. The first studies on MPL for CSC used an 810 nm diode laser as the laser source [15, 16, 18]. Subthreshold micropulse yellow wavelength (577 nm) laser (SMYL) is a newer technology that offers major advantages such as peak absorption of oxyhemoglobin, minimal xanthophyll absorption in the macula, and better penetration [19]. The aim of this study was to compare the efficacy and safety of SMYL and low-fluence PDT in the treatment of chronic CSC. This is the first study that compares the treatment outcomes of SMYL and PDT in patients with chronic CSC.

## 2. Methods

This retrospective comparative case series included 33 eyes of 30 patients with chronic CSC treated with either low-fluence PDT (18 eyes) or SMYL (15 eyes) therapy at the Ankara University Faculty of Medicine, Department of Ophthalmology, between January 2012 and January 2015. The study was conducted in accordance with the Declaration of Helsinki.

The diagnosis of chronic CSC was confirmed by clinical examination, spectral domain optical coherence tomography (SD-OCT) (Spectralis; Heidelberg Engineering, Inc., Heidelberg, Germany), fundus fluorescein angiography (FA), indocyanine green angiography (ICGA), and fundus autofluorescence (FAF) imaging (Heidelberg Retina Angiograph 2; Heidelberg Engineering, Heidelberg, Germany).

Inclusion criteria were as follows:

1. Visual impairment history lasting at least six months.
2. CSC with SRF involving the fovea and documented by SD-OCT.
3. Presence of single or multiple active leakage sites and/or RPE changes on baseline FA.
4. Absence of prior history of micropulse laser or PDT therapies.
5. Follow-up period of at least 12 months after both treatment modalities.

There were not any criteria such as disease severity for selection of the treatment modalities. Patients with a history of comorbid ocular conditions such as age-related macular degeneration, diabetic macular edema, advanced glaucoma, optic neuropathy, or intraocular surgery within the previous six months were excluded.

In the micropulse treatment group (15 eyes), SMYL treatment (Supra Scan 577; Quantel Medical, Clermont-Ferrand, France) had been performed by the same experienced surgeon (EO), using the following parameters: low-intensity (5% duty cycle) and high-density (confluent spots) treatment with 200 msn duration and 160 *μ*m spot size on the slit-lamp adaptor. The power was initially increased upward to the minimum threshold value to cause a barely visible burn on micropulse mode, and then it was adjusted to half of that value. An OCT-guided approach was preferred, and in this manner, an appropriate scan shape was chosen to cover the edematous area entirely on the OCT thickness map.

In the low-fluence PDT group (18 eyes), verteporfin (Visudyne; Novartis, Basel, Switzerland) was administered intravenously at a dose of 6 mg/m^2^ over ten minutes. Fifteen minutes after the start of the infusion, a 689 nm laser was delivered for 83 seconds at a reduced light dose of 25 J/cm^2^ and an intensity of 300 mW/cm^2^. The same experienced specialist (FB) performed the procedure on all of the patients in this group. The PDT application location was based on choroidal hyperpermeability identified by ICGA. After the treatment, the patients were instructed to avoid sunlight exposure for five days.

All of the patients were examined at the baseline visit and one, three, six, nine, and 12 months after undergoing the SMYL or PDT therapy. At each visit, each participant underwent a complete ophthalmic examination that included best corrected visual acuity (BCVA) with Early Treatment Diabetic Retinopathy Study (ETDRS) charts, slit-lamp biomicroscopy, intraocular pressure measurements, dilated fundus examination, FAF imaging, and OCT; FA was repeated as needed. The morphologic results of the treatment were evaluated with SD-OCT in terms of SRF height and central macular thickness (CMT). Subretinal fluid height was measured manually between the outer segment of the photoreceptor layer and the apical face of the RPE layer.

The patients were divided into three groups according to response to treatment: complete response, incomplete response, and unresponsive to therapy. Complete response was defined as complete resolution of SRF in the central 1000 micron area. Any change in SRF within ±100 *μ*m was defined as unresponsive. If the SRF decreased more than 100 *μ*m but was not fully resorbed, the treatment response was defined as incomplete resolution.

The main outcome measures were changes in BCVA and SRF height between the baseline and follow-up examinations. The second outcome measure was to compare the complete anatomic resolution, recurrence, and complication rate between treatment modalities at the end of the 1 year.

Data are shown as mean and standard deviation for continuous variables. For visual acuity assessment, BCVA was converted to logarithm of the minimum angle of resolution (logMAR) equivalents; in addition, ETDRS letters were used in the statistical analysis. Statistical analysis was performed using SPSS software for Windows version 15.0 (SPSS, Inc., Chicago, IL). A *p* value <0.05 was considered statistically significant. Specific differences in mean values between different treatment groups were tested for significance using Mann-Whitney *U* test with Bonferroni's correction of *p* values for multiple comparison. The changes in the same group throughout the follow-up visits were compared using the Friedman test. Kruskal-Wallis test was used for the evaluation of singly ordered categorical data.

## 3. Results

Thirty-three eyes of 30 patients with chronic CSC were analyzed in this study. The patient group included 22 (73%) male patients and eight (27%) female patients. The mean age (±SD) of the patients at presentation was 49.9 ± 11.1 (31–73) years. The PDT group was older than the SMYL group (52.7 ± 11.2 years versus 44.7 ± 9.5, resp.; *p* = 0.037), and the duration of chronic CSC prior to treatment was longer in the PDT group (18.8 ± 13.5 months versus 13.0 ± 9.1 months, resp.; *p* = 0.330). None of the patients in the SMYL group underwent previous therapy. Ten patients in the PDT group had previously received intravitreal ranibizumab injections, but the last injection was performed at least three months prior to PDT. The mean number of ranibizumab injections was 4.7 ± 3.2.

**Figure 1:**
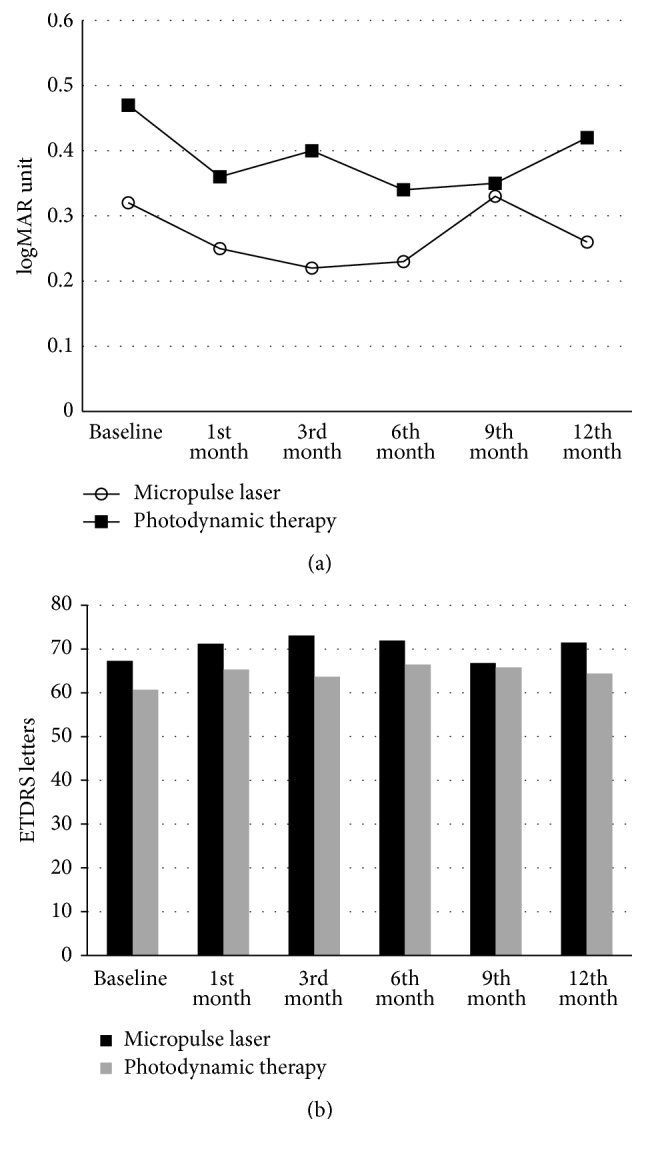
The changes in logMAR visual acuity scores (a) and ETDRS letters (b) throughout follow-up were shown.

Initial mean visual acuity was 0.47 ± 0.32 logMAR units in the PDT group and 0.32 ± 0.29 logMAR units in the SMYL group (*p* = 0.126). All patients had at least a 12-month follow-up after treatment. In the PDT group, BCVA improved at least 5 ETDRS letters in six eyes (33.3%) and remained stable (within ±4 ETDRS letters) in six eyes (33.3%) (Table 1). In the SMYL group, BCVA improved at least 5 ETDRS letters in ten eyes (66.7%) and remained stable in one eye (6.7%) (*p* = 0.101). Mean BCVA improved from 67.3 ± 14.2 to 71.5 ± 21.4 ETDRS letters in SMYL group and improved from 60.7 ± 16.3 to 64.4 ± 24.9 ETDRS letters in PDT group (*p* = 0.285, *p* = 0.440). The changes in logMAR visual acuity scores and ETDRS letters throughout the follow-up period are shown in Figure 1; there were no statistical differences between the groups (*p* = 0.079 for both groups).

**Figure 2:**
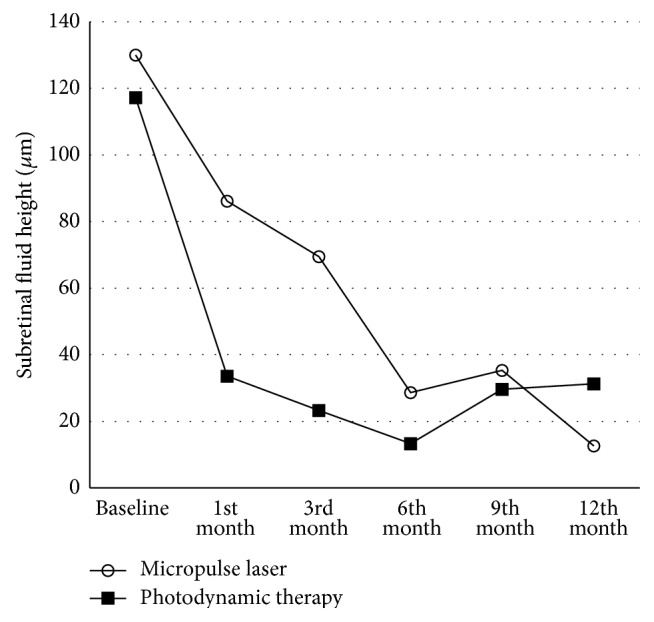
The changes in subretinal fluid height throughout follow-up were shown.

Mean SRF height decreased from 117.2 ± 59 *μ*m to 31.3 ± 57 *μ*m in the PDT group and from 130.0 ± 105 *μ*m to 12.6 ± 21 *μ*m in the SMYL group. The reduction in SRF from baseline to the 12th month was statistically significant in both groups (*p* = 0.031 and *p* = 0.014, resp.). The changes in SRF height throughout the follow-up period are shown in Figure 2; there were no statistical differences between the treatment groups (*p* = 0.735). Mean CMT decreased from 242.9 ± 80 *μ*m to 156.9 ± 61 *μ*m in the PDT group and from 287.3 ± 126 *μ*m to 138.0 ± 41 *μ*m in the SMYL group (*p* = 0.098 and *p* = 0.003, resp.). There were no statistical differences between the treatment groups throughout follow-up (*p* = 0.338).

**Figure 3:**
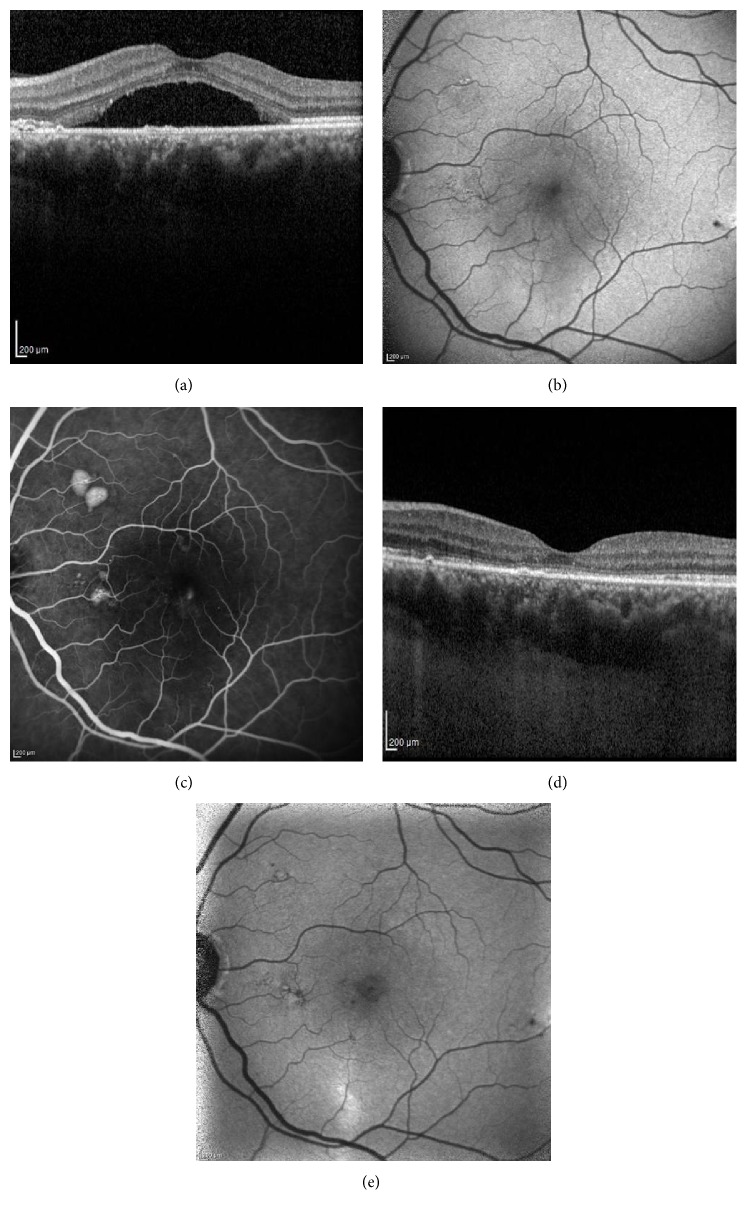
Spectral domain optical coherence tomography (SD-OCT), fundus autofluorescence (FAF), and fluorescein angiography (FA) images of a 47-year-old female patient with CSC for 7 months. Left eye before photodynamic therapy (PDT): chronic subretinal fluid on SD-OCT (a), hypoautofluorescence on FAF image due to the blockage effect of the subretinal fluid (b), and focal leakage of fluorescein and two small pigment epithelial detachments at the late phase of FA (c). Eight months after PDT treatment: complete resolution of the subretinal fluid (d) and disappearance of hypoautofluorescence on FAF (e).

**Figure 4:**
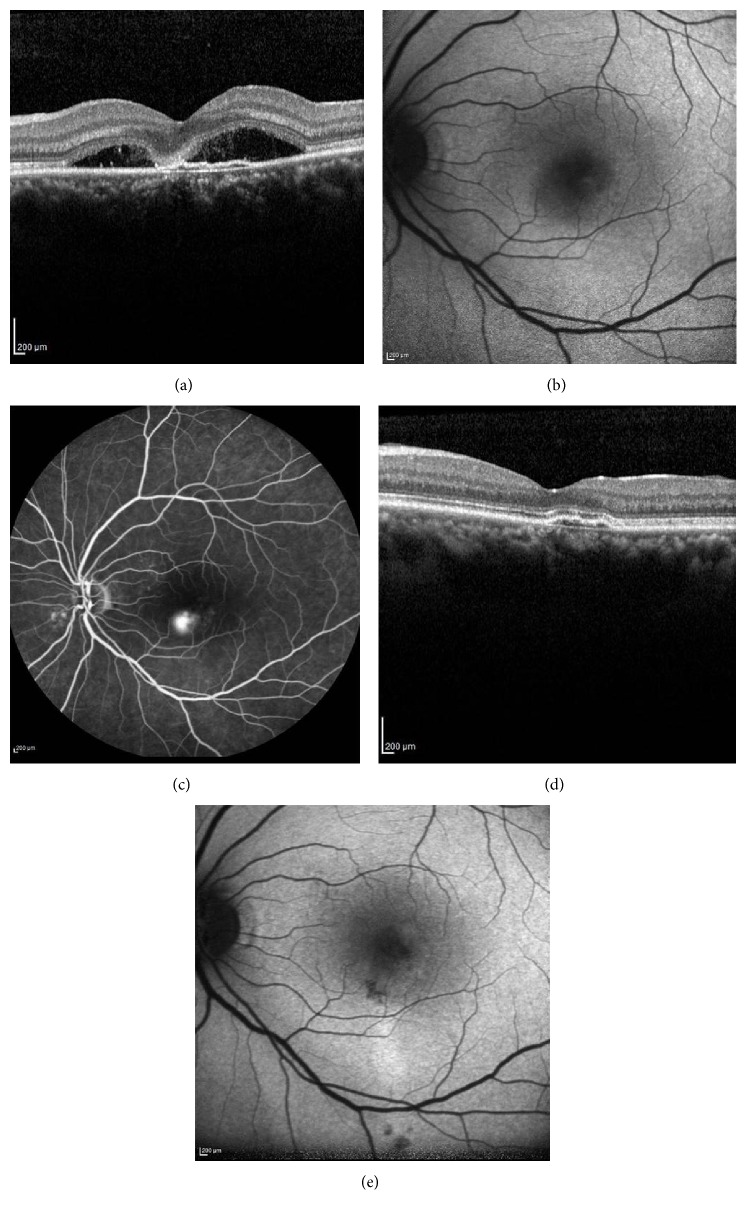
Spectral domain optical coherence tomography (SD-OCT), fundus autofluorescence (FAF), and fluorescein angiography (FA) images of a 42-year-old female patient with CSC for 7 months. Left eye before subthreshold micropulse yellow wavelength laser (SMYL) treatment: chronic subretinal fluid with fibrin accumulation on SD-OCT (a), increased hypoautofluorescence on FAF image due to the blockage effect of the subretinal fluid (b), and focal leakage of fluorescein at the late phase of FA (c). Nine months after SMYL treatment: complete resolution of the subretinal fluid (d) and hypoautofluorescence spots due to RPE atrophies at the previous leakage area on FAF (e).

Treatment responses based on SRF resolution were evaluated at the 12-month follow-up (Table 2). In the PDT group, 13 eyes (72.2%) achieved complete anatomic resolution of SRF in the central macula (Figure 3) and one eye (5.6%) had incomplete resolution, confirmed by OCT. Recurrence of SRF after complete resolution occurred in one eye (5.6%); three eyes (16.7%) were unresponsive to PDT. Of these unresponsive eyes, one eye received one more seance of PDT; however, the patient was unresponsive to the treatment again. The other patients were not retreated. In the SMYL group, 12 eyes (80.0%) achieved complete anatomic resolution of SRF in the central macula (Figure 4) and one eye (6.7%) had incomplete resolution. Subthreshold micropulse yellow wavelength laser treatment was repeated in this eye. However, complete anatomic response could not be achieved again. Recurrence of SRF after complete resolution occurred in two eyes (13.3%); no eyes were unresponsive to SMYL. There was no statistical difference between the PDT and SMYL groups in terms of treatment response (*p* = 0.486). Fundus autofluorescence imaging used to determine the safety profile of the SMYL group revealed hypofluorescent laser spots that were not visible in the fundus examination in one case. There were no significant side effects in the PDT group.

## 4. Discussion

In the present study, the treatment outcomes of chronic CSC patients treated with either low-fluence PDT or SMYL were compared, and changes in logMAR visual acuity scores, ETDRS letters, SRF height, and CMT throughout the follow-up were analyzed. To our knowledge, this is the first study in the literature to compare SMYL and low-fluence PDT treatment results in chronic CRC patients. The results of the study indicate that there were no statistically significant differences in the parameters listed above between the two treatment modalities. Subthreshold micropulse yellow wavelength laser seems to be an effective modality in treatment response, visual acuity improvement, and SRF reduction.

The exact pathophysiologic changes that lead to the characteristic RPE leakage associated with CSC remain unclear. However, ICGA has demonstrated the effect of choroidal circulatory abnormalities in the development of CSC, such as hyperpermeability from choriocapillaris [20], venous dilation [21], and vascular congestion [22]. In the treatment of CSC, the aim is to induce absorption of SRF and to improve or stabilize visual acuity.

Photodynamic therapy with verteporfin has proven to be effective according to several reports [11, 23, 24]. The mechanism of action of PDT depends on transient choroidal hypoperfusion leading to choroidal vascular remodeling and the reduction of choroidal exudation [6, 23, 25]. However, the conventional PDT protocol has several potential serious adverse effects, including RPE changes, permanent choroidal ischemia, and secondary choroidal neovascularization [11, 12, 23]. To reduce the risk of these potential adverse effects, modified PDT protocols have been developed, such as half-dose verteporfin administration [13, 26, 27] and low-fluence rate [28, 29]. It has been reported that low-fluence PDT reduces choroidal hypoperfusion without causing significant differences in treatment efficacy [28]. Photodynamic therapy is a relatively invasive procedure that requires the intravenous injection of verteporfin. Also, ICG may cause anaphylactic and urticarial reactions in patients both with and without a history of allergy, and hepatic diseases, hemodialysis, and pregnancy are relative contraindications for ICG [30]. A low-fluence PDT protocol was used in the present study, with complete resolution of SFR achieved in 72.2% of the patients.

The subthreshold MPL technique introduced the term “photostimulation,” as opposed to “photocoagulation” [18]. The mechanism depends on low-intensity, high-density laser applications in envelopes of repetitive short pulses [31]. These pulses stimulate the production of intracellular antiangiogenic and restorative biological factors without causing visible laser scars [32, 33]. Wavelengths of either 810 or 577 nm can be used as the laser source in micropulse photostimulation. This technique has been used with promising results in the treatment of macular edema in diabetic retinopathy and branch retinal vein occlusion [34–36]. Chen et al. [15] studied subthreshold diode MPL in 26 eyes of 25 CSC patients and reported it to be effective in the presence of point source leakage. However, a less favorable response was noted in eyes with diffuse leakage. Another study reported that, after ICG dye-enhanced subthreshold MPL, neuroepithelial detachment was completely resolved in five of seven patients with CSC within four to eight weeks [16]. In a recent study comparing the outcomes of subthreshold MPL and intravitreal bevacizumab injections, subthreshold MPL photocoagulation was found to be superior to intravitreal injections of 1.25 mg bevacizumab in the treatment of CSC [18]. All of these promising findings define subthreshold MPL as a possible treatment option for chronic CSC.

Subthreshold micropulse yellow wavelength laser is a quite different modality than subthreshold micropulse diode laser. The yellow wavelength (577 nm) laser produces combined absorption by both melanin and oxyhemoglobin, which leads to maximum absorption in the pigment epithelium and choriocapillaris [37]. It also has negligible xanthophyll absorption, which allows repeated treatment close to the fovea [19]. While the longer wavelength provides better penetration, it also leads to energy concentration in a smaller volume and allows a shorter pulse duration. The major advantages of the procedure are the tissue-sparing effect and repeatability of the sessions after three to six months in case of recurrence of SRF or unresponsiveness to therapy. Subthreshold micropulse yellow wavelength laser might also lead to an increase in retinal sensitivity in the macular area [38]. In the present study, the effects of the SMYL therapy on macular sensitivity could not be evaluated, due to the lack of availability of a microperimetry device. However, we believe SMYL therapy may improve retinal sensitivity in addition to increasing visual acuity.

Only two studies in the literature have reported the efficacy of SMYL in chronic CSC patients. The first study included 15 eyes of 13 patients who had CSC for more than three months. The mean follow-up period was eight weeks, and the researchers reported a reduction in mean SRF height from 232 to 49 *μ*m at the final visit. They determined that the procedure was safe and that it caused no obvious changes in RPE as seen on OCT and FAF [38]. In the second study, the authors reported the results of ten eyes of ten chronic or chronic recurrent CSC patients who received SMYL with a 15% duty cycle. The logMAR visual acuity scores improved from 0.21 ± 0.21 to 0.035 ± 0.063 at the end of the follow-up period (mean, 8 months; range, 3–18 months) [39]. The present study is the first study to compare the treatment outcomes of low-fluence PDT and SMYL, with follow-up period of 12 months. In this study, 80% of the eyes in the SMYL group achieved complete resorption of SRF (72.2% in PDT group), and all of the eyes were responsive to therapy. However, two patients experienced recurrence of CSC, and in another patient, FAF revealed hypofluorescent laser spots that were not visible in the fundus examination. Retinal laser spots on FAF could occur in rare circumstances as a result of RPE changes. The occurrence of retinal burn depends on the pigmentation of the individual and the applied laser power. Several methods were developed for MPL power adjustment. Some authors adjust laser power upward to the minimum threshold value for a visible burn in a continuous wave mode and then switch the apparatus to micropulse model [15, 38, 40]. Other researchers have also used the micropulse mode for power titration and applied 50–80% of the minimum threshold power to cause a barely visible burn [39, 41]; that power adjustment method was used in the present study. However, further studies are needed to determine a standard power titration protocol in SMYL applications.

The major limitation of the present study is the retrospective design. A randomized prospective controlled trial will be more rational to compare the treatment modalities. Also the longer duration of the disease and the presence of eyes previously treated with intravitreal anti-VEGF therapy in PDT group are the other main limitations of the study.

In conclusion, SMYL seems to be effective in providing resorption of SRF in the treatment of chronic CSC. Although not statistically significant, the rate of complete resolution of SRF and ≥ 5 increase in ETDRS letters were higher in SMYL group. Also, in contrast to the PDT group, no eyes were unresponsive to SMYL. Major advantages of SMYL are having a tissue-sparing effect, being a noninvasive procedure, and allowing safe repetition of the therapy after three to six months. In addition to improving BCVA, SMYL may also improve retinal sensitivity in the macular area without causing collateral chorioretinal damage. Further prospective studies are needed to investigate the correlation between changes in visual acuity and macular sensitivity on microperimetry after SMYL in the treatment of CSC.

## Figures and Tables

**Table 1:** Final best corrected visual acuity changes in low-fluence photodynamic therapy and subthreshold micropulse yellow laser therapy groups.

BCVA change	Low-fluence PDT *n* (%)	SMYL *n* (%)
Increase ≥ 5 ETDRS letters	6 (33.3%)	10 (66.7%)
Stable (within ±4 ETDRS letters)	6 (33.3%)	1 (6.7%)
Decrease ≥ 5 ETDRS letters	6 (33.3%)	4 (26.7%)

Total	18	15

		*p* = 0.101

**Table 2:** Treatment responses based on SRF resolution in low-fluence photodynamic therapy and subthreshold micropulse yellow laser therapy groups at the end of one-year follow-up period.

Treatment response	Low-fluence PDT *n* (%)	SMYL *n* (%)
Complete resolution of SRF	13 (72.2%)	12 (80.0%)
Incomplete resolution of SRF	1 (5.6%)	1 (6.7%)
Unresponsive	3 (16.7%)	None
Recurrence	1 (5.6%)	2 (13.3%)

Total	18	15

		*p* = 0.486
